# Collaborations Between IPUMS and Genealogical Organizations, 1999–2022

**DOI:** 10.51964/hlcs12920

**Published:** 2023-05

**Authors:** Steven Ruggles

**Affiliations:** University of Minnesota

**Keywords:** Census data, Genealogy, IPUMS

## Abstract

From 1999 to 2019, IPUMS collaborated with genealogical organizations to develop massive individual-level census datasets spanning the 1790 through 1940 period, and we are currently working on the 1950 census. This research note describes how our genealogical collaborations came about. We focus on our collaborations with the Church of Jesus Christ of Latter-Day Saints Family and Church History Department (later known as FamilySearch) and the private genealogical companies HeritageQuest and Ancestry.com.

## 1. INTRODUCTION

Since 1999, the Integrated Public Use Microdata Series (IPUMS) has collaborated with genealogical organizations to digitally transcribe full-count censuses that will soon cover more than 800 million individual-level census observations spanning the period from 1850 to 1950. The availability of these data creates unprecedented opportunities to construct a longitudinal panel tracing individuals across their lives and families over generations. In 2021, IPUMS released the Multigenerational Longitudinal Panel (IPUMS-MLP) linking individuals across the censuses of 1850 through 1940 (Helgertz et al., 2021). We are now enhancing IPUMS-MLP by incorporating information from the Social Security Administration that includes name changes, helping us to link women who change their names upon marriage. Planned future improvements of IPUMS-MLP include links to death records, military records from the world wars, and linkages to more recent censuses and surveys. The development of this massive resource for life-course studies would have been impossible to contemplate without a series of collaborations with genealogical partners.

In its early years, IPUMS focused on sample data rather than full-count censuses. Between 1989 and 1999, IPUMS created 1-in-100 samples of all the U.S. censuses for which the individual-level census manuscripts were then available. We started with the 1880 census, and then did samples of the censuses of 1850, 1920, 1860, and 1870. Samuel Preston had already developed samples of the 1900 and 1910 censuses, but they were comparatively small so we began work in 1998 to expand those samples (Ruggles, 2005).

To transcribe the data from microfilm into machine-readable form, we had a staff of full-time professional data-entry operators, at times numbering up to a dozen. Graduate research assistants were responsible for consistency checking and data cleaning, and for building the data dictionaries needed to convert the censuses responses — which were captured as literal string transcriptions of open-ended census responses — into numerically-coded standard classifications. I developed the necessary software in collaboration with Todd Gardner, a talented graduate assistant. In 1992 I designed a metadata-driven system to harmonize the data over time, and in 1995 Gardner implemented a web-based data access tool to create customized pooled data extracts.

For the next two decades, from 1999 to 2019, IPUMS collaborated with genealogical organizations to develop larger samples and complete individual-level census enumerations spanning the 1790 through 1940 period. That effort was completed on September 25, 2019 with the release of the last two full-count censuses for 1860 and 1870. IPUMS now disseminates full-count data spanning the period 1790 to 1940, including household-level enumerations of the pre-1850 period (Ruggles, 2014). We are now working on a new collaboration to develop a full-count dataset for the 1950 census.

This research note describes how our genealogical collaborations came about. We focus on our work in the United States with the Church of Jesus Christ of Latter-Day Saints Family and Church History Department (later known as FamilySearch) and the private genealogical companies HeritageQuest and Ancestry.com.^1^

## 2. THE 1880 CENSUS COLLABORATION

When surfing the net on Alta Vista one day in early May of 1999, I came across a bulletin board with a posting from someone who had volunteered to do data-entry of the 1880 census for the Church of Jesus Christ of Latter Day Saints (LDS). The post simply noted that they had completed their assignment to transcribe the data from a particular set of reels.

The Genealogical Society of Utah was established in 1894 by the LDS church for the purpose of sharing information and educating the public about genealogy. Church members are encouraged to identify deceased relatives who did not receive ordinances of salvation during their lifetimes. Members can then perform the ordinances on behalf of those relatives by proxy. To enable genealogical research, the Society collected vital records, census listings, and other genealogical sources. In 1938 the Society began microfilming these sources and disseminating them through a network of family history libraries around the world (FamilySearch, 2018). Digital transcriptions of genealogical sources were a logical extension of this work.

I was already aware that the LDS had overseen a volunteer project to transcribe the 1881 census of England and Wales. The British censuses were copyrighted by Her Majesty's Stationers Office, and to get the rights to disseminate the British census data to genealogists the LDS had to meet the conditions of the crown, and one of those conditions was to deposit a copy of the data with the History Data Service of the U.K. Data Archive, which was working hard to make the data usable for quantitative analysis (Woollard, 2000).

There had been no hint that a parallel project was underway in the United States. I began a process of cold-calling people in the Church to see if I could determine what was going on. The LDS did not have a publicly accessible staff directory, so I found names and numbers for various people who worked on family history for the Church in various recesses of the internet. Most of the people I reached either had no idea what I was talking about or did not want to discuss it, but after a week and a dozen calls someone suggested I contact Ray Madsen, who was Manager of Resource Files in the Family and Church History Department.

Madsen acknowledged that the Church was nearing completion of a massive project to transcribe information for all 50 million people in the 1880 U.S. census. They had been working since 1982, and over the next 17 years, more than 1,000 volunteers contributed 11.5 million hours to the effort, keying data that describe 50.5 million persons residing in 11 million households (Deseret News, 2001).

I was excited, and tried to explain to Madsen what a valuable resource the data would be for historical research. He was initially skeptical. I made things worse by offering him a large sum of money for the data, which I felt confident I could get from funding agencies. Madsen seemed insulted by the offer, explaining that the church was not a commercial operation and this work was being done for higher purposes.

Madsen eventually revealed that they were having a great deal of trouble managing the data. Volunteers had entered data over an 18-year period on microcomputers using two different data entry programs, and the files were a mess. I volunteered to fix it, and eventually managed to convince Madsen I knew what I was doing. In mid-May we reached a tentative agreement to clean, organize, and document the data in exchange for the right to disseminate it to the academic community.

In June 1999, the LDS provided us with 66,000 cases of 1880 data drawn from two microfilm reels covering parts of Arkansas and Massachusetts so that we could carry out a pilot study for the cleaning project. The Arkansas and Massachusetts data were created using different data-entry software. For the first decade of data entry, the LDS used a program called the “Volunteer Data Entry System” (VDE), and thereafter they used the “Universal Data Entry System” (UDE). 60% of the cases were entered using the VDE and the remainder using the UDE. There were significant differences in format and data processing errors under the two systems, and our cleaning procedures had to account for both VDE and UDE.

In preparation for the creation of a genealogical look-up system, the LDS had converted the raw VDE and UDE data into Oracle database format and carried out a variety of edits to make the two databases internally consistent and compatible with one another. Some of this work was labor-intensive, involving manual examination of millions of cases with invalid entries. Other aspects of the cleaning process were automated, such as the elimination of duplicate cases. Unfortunately, the cleaning work was badly flawed, the LDS lost about 10% of cases. Moreover, the new database inadvertently dropped two key variables: batch number, needed to uniquely identify each case, and the new dwelling flag, which identifies the beginning of each dwelling. When these errors were discovered, the LDS went back to the raw data and created a new Oracle database that included the missing information. Thus, by June 1999 there were two versions of the database: an “original” version, which was nearly complete but which has not yet been cleaned, and a “processed” version, which was higher quality but was missing cases and key variables. Since the unique identifiers were dropped from the processed version of the file, it was non-trivial task to merge the two files.

On July 19 and 20, 1999, the LDS sent a delegation to Minneapolis to inspect our operation and discuss the details of our proposed data cleaning strategy (R. Madsen, personal communication, July 17, 1999). They were favorably impressed, and we reached a formal agreement with the Church, which was ratified in the fall by the 90 Brethren and the 12 Apostles of the Church. We agreed to an ambitious delivery date of December 13, 2000 for the complete cleaned data.

In the meantime, I submitted proposals to NSF in August and NIH in October to fund the work. Both were successful; indeed, the NIH proposal scored in the top 0.3% of proposals. With funding reasonably assured, we began ramping up production in November 1999.

We completed the cleaning on time, and turned to the work of coding needed to convert the data into a form suitable for analysis. The LDS released their version of the database on 56 CD-ROM disks in July 2001. In 2002 the LDS honored us with a handsome plaque “in recognition of their exceptional contribution to the development of the 1880 United States Census on Compact Disk.” At that time, Ray Madsen assured me that LDS would never again get involved in a similar project, since it had been so much trouble.

We released our first version of the full-count 1880 data via the North Atlantic Population Project website in July 2003. We continued working on the 1880 LDS data until 2009. The LDS had not entered all the information on the form, so our data-entry staff entered the missing variables for a 10% sample of the cases. We also developed the IPUMS Linked Representative Samples, which linked individuals from the full-count 1880 census to each of the IPUMS 1% samples to provide two observations for each linked case.

## 3. 1930 IPUMS COLLABORATION

Under the 72-year privacy rule governing the U.S. Census, the census manuscripts of the 1930 census were released on April 1, 2002. In anticipation of that event, we submitted a proposal to NIH to create a 1% 1930 IPUMS sample in January 2001. When our proposal for a new sample of the 1900 census had been reviewed in 1998, a reviewer had chided us for neglecting to explore new technologies to speed data entry. Accordingly, our 1930 proposal included several data-entry innovations. The most important of these was keying data from digital images of the forms instead of from microfilm. We argued that this would simplify sampling, eliminating an initial pass through the microfilm to determine the page sequences on each reel. The data-entry operators could load the needed pages instantly without scrolling through unwanted material. Moreover, the process would be more ergonomic than the traditional microfilm readers.^2^

We required a source of images of the 1930 census manuscripts, so we solicited bids from the two genealogical organizations that had announced that they planned to produce digital images of the 1930 census: Heritage Quest (a division of ProQuest Information and Learning) and Ancestry.com (which was then a division of MyFamily.com).

When I contacted Spencer Woolley, Director of Electronic Production at Ancestry.com, he expressed amazement that we were doing our own data entry in house. He explained that Ancestry.com had hundreds of highly skilled data-entry staff, and that they were already digitizing many of the fields we planned to enter. He said that Ancestry.com could add the extra fields we needed for a small fraction of what it would cost if we did it ourselves. Accordingly, we drew up the specification for exactly what we needed, and solicited bids from both MyFamily.com and Heritage Quest, which was also creating a 100% index of the 1930 census.

After a lengthy process of hammering out sample designs, error tolerances, and the like, we received responses from both vendors. Our request for proposals specified a 5% sample instead of the 1% sample we had specified in the grant.

MyFamily.com came in at $700,000 and Heritage Quest came in at $475,000 (J. Gehring, personal communication, April 12, 2002; S. Woolley, personal communication, April 25, 2002). We went with Heritage Quest both because of the price and because they seemed to have a better understanding of what we were up to. The contract for professional services with Heritage Quest did not require facilities and administration charges, so our indirect cost budget declined by some $225,000; we were able to repurpose those funds, which gave us sufficient funding to keep our full retinue of data entry staff employed on the project, checking the work by Heritage Quest and cleaning the data they produced.

Heritage Quest outsourced data entry to Bangladesh. Ironically, in June 2003 Heritage Quest reached an agreement with Ancestry.com to avoid duplication of data-entry effort, and as a result Heritage Quest began contracting with Ancestry for data entry. Accordingly, data entry for the 1930 project switched to Ancestry's data-entry vendor, Bejing Formax based in Zhongguancun Science Park, the “Silicon Valley of China” (J. Gehring, personal communication, June 3, 2003).

At about the same time, IPUMS received funding to expand the 1900 sample from 1% to 6%, using the same outsourcing approach as 1930. All data entry for the 1900 expansion project was carried out by Beijing Formax under the Ancestry.com contract with Heritage Quest. That project was completed in 2008.

## 4. 1850 FAMILYSEARCH COLLABORATION

After the first phase of the 1880 project was complete, we continued to have discussions with Ray Madsen about various potential projects involving Norwegian data, mortality records, and other topics. In 2007 a contingent from IPUMS visited the Family and Church History Department in Salt Lake City to discuss the potential for a mortality project.

That project never came to pass, but in the course of the meeting the LDS staff presented information about a new crowdsourcing project. They had developed a web-based application for data entry, and were soon to begin entering data from additional censuses. The user-friendly software presented an image of a manuscript census form on the top half of the screen and a data-entry form on the lower half and provides guidance to data-entry volunteers as they move from field to field transcribing records. The effort was extraordinarily successful.

By 2009, just three years after FamilySearch launched the system, the project had attracted 100,000 volunteers who transcribed 250 million records (Deseret News, 2009). To maximize accuracy, two volunteers independently keyed each entry, and a third volunteer arbitrated discrepancies. They finished the 1850 census first. Building on the success of the 1880 project, IPUMS reached an agreement to improve the FamilySearch version of the 1850 census through data cleaning and adding variables that had been omitted by the digitization project. That project was completed in 2015.

By July 2011, FamilySearch had transcribed data from all the publicly accessible U.S. censuses from 1790 to 1930. Unfortunately, by then our friend Ray Madson had retired, and LDS lost interest in collaborating with us.

## 5. 1940 ANCESTRY.COM COLLABORATION

In March 2009 I was on a study section for the National Institute on Aging, and the late Richard Suzman approached me during a coffee break to ask me about doing an index of the 1940 census using great recession stimulus funds available under the American Recovery and Reinvestment Act. He was interested in 1940 so that researchers could get information about early life conditions for survey respondents. I asked for cost estimates from the National Archives and Records Administration (NARA), and it looked like they could probably do it within the scope of the available funding. They were enthusiastic; the British National archives had done the same thing for the 1901 census a few years before, and it was a terrific “crashing” success (BBC, 2002).

But the deadlines were tight, and at that moment NARA did not have a permanent director, so I could not get a quote in time. The next year, NARA and Census held a workshop on scientific uses of the 1940 census (NARA, 2010). At the workshop, I complained bitterly about the lost opportunity the year before. A month later, I got a call from Todd Godfrey, the Vice President for Global Content at Ancestry.com.

It turned out Godfrey had heard me speak at the 1940 workshop, and he was calling to find out if there was any way we could collaborate on digitizing the 1940 census. Ancestry.com had planned to transcribe the basic census questions needed by genealogists: name, age, sex, marital status, and birthplace. MPC made an agreement with Ancestry.com to share the additional costs needed to transcribe virtually the entire census form, and make the full census freely available for scientific research and education.

We raised money from NSF, NIA, and NICHD to subsidize data entry of the fields with no genealogical interest, like income and education. With 132 million person records and 70 variables, the 1940 census database is the largest data collection from a single census ever made freely accessible for scientific research. Like our 1930 and 1900 large samples, the data entry was done by off-shore vendors under contract with Ancestry.com, mainly Beijing Formax, the same outfit that had transcribed our large samples of 1930 and 1900.

## 6. BIG MICRODATA

In late 2012, some six months after the 1940 project began, Godfrey called and suggested that we do the same thing for the censuses of 1860 to 1930 which Ancestry.com had transcribed through outsourcing (Ancestry.com, 2006). We were thrilled by this idea, and entered into a long negotiation.

One potential complication was introduced by a collaboration between Ancestry.com and Familysearch. In July 2008, FamilySearch and Ancestry reached an agreement to merge their indexes for the historical censuses of 1900 to 1930 (Ancestry.com, 2008). For the 19th century census years, FamilySearch volunteers transcribed the data twice, and arbitrated the discrepancies between the two transcriptions. Under the new agreement, FamilySearch used the Ancestry.com version of the data as their verification copy, so they only had to enter the data once. Ancestry.com benefitted from the arrangement because they got more accurate, verified data; FamilySearch benefitted because they only had to enter the data once.

When they made their agreement, there was a crucial little clause. My former student Lisa Dillon at the University of Montreal had worked with Ray Madsen to make the 1881 census of Canada accessible for scientific research. When she got wind of the Ancestry-LDS collaboration, she was worried that being a commercial company, Ancestry would block access to the data by academic users. So she convinced Ray to insert a clause that either LDS or Ancestry had the right share the data with the MPC for dissemination to the scholarly community (Dillon & Ruggles, 2001).

It turned out that it was Ancestry, not LDS as Dillon had assumed, that wanted to share. Because of the “Dillon clause”, Ancestry.com had the rights to give us data that had been entered by FamilySearch, and there was no need to negotiate any additional permissions. Accordingly, in March 2013 the University of Minnesota signed an agreement with Ancestry.com to make the merged data collections available for scientific research and educational purposes. In addition, they gave us all of their other U.S. censuses, including the complete censuses of 1860, 1870, and the household-level data for 1790 to 1840.

The microdata from 1860 to 1930 did not yet include every variable that was originally enumerated; Ancestry and FamilySearch focused mainly on the variables most useful for genealogical research. The digital files for all census years included a core set of variables valuable for demographic research, including geographic location, age, sex, race, marital status, relation-to head, birthplace, and the birthplace of each individual's mother and father, allowing the identification of second-generation Americans. Other key variables — such as year of immigration, duration of marriage, literacy, occupation, children ever born, children surviving, and disability — were available sporadically.

In March 2014, the University of Minnesota signed an agreement to fill in virtually all the remaining variables in the 1850–1930 data through new data entry. Under the terms of the agreement, Ancestry. com covered about 75% of the cost and IPUMS covered 25%. The last files were released in September 2019.

## 7. 1950 COLLABORATION

In June 2019 we began negotiations with Ancestry.com to develop a full-count dataset for the 1950 census. Under the 72 year rule, the 1950 census enumeration forms were scheduled for release on April 1, 2022, and we wanted to leave plenty of time to raise funding for the project. Ancestry was investigating the feasibility of optical character recognition to reduce the cost of digitization, and was simultaneously in negotiation with FamilySearch about a possible collaboration.

After extensive discussions we reached an agreement with Ancestry to collaborate on the 1950 census in January 2021, and at about the same time Ancestry also reached an agreement with FamilySearch. The project is currently underway with a three-way division of labor. Ancestry is producing the base files using optical recognition software. FamilySearch is using volunteer crowdsourcing to verify and correct the machine transcription. IPUMS is contributing data quality evaluations and variable classifications, as well as a financial subsidy to underwrite digitization of the non-genealogical variables. We anticipate that a preliminary version of the data will be available in 2023.

## 8. CONCLUSION

The opportunities for IPUMS to collaborate with FamilySearch, Ancestry.com, and Heritage Quest were largely fortuitous. Our collaboration with the LDS began when I stumbled across an Internet posting about the 1880 census transcription while surfing the net. The Heritage Quest collaboration was stimulated by a random comment by a proposal reviewer. Our collaboration with Ancestry began with an idea of Richard Suzman for an effective use of economic stimulus funds on a 1940 index, which led to my whining presentation at a meeting that happened to be also attended by the key person at Ancestry.com. Much has to do with being in the right place at the right time. As Herbert Fisher (1936, p. lx) expressed it, historians should “recognize in the development of human destinies the play of the contingent and unforeseen.”

The other big factor in the success of these collaborations has been the ability to raise substantial funding very quickly. Without the two decades of continuous support from the National Science Foundation, the National Institute of Child Health and Human Development, and the National Institute on Aging, these genealogical collaborations would have been impossible.

The explosion of digital transcriptions by genealogical organizations worldwide has already had transformational impact on historical demography by slashing the cost of creating massive new databases. The development of automatic handwriting recognition technology can be expected to reduce the cost of digital transcription further, and it is reasonable to anticipate that virtually every historical source that includes names and other identifying characteristics will be converted into digital form in the foreseeable future. Linked together, these sources will provide unprecedented opportunities to examine historical life courses. To successfully exploit the new bounty of data, historical demographers must find ways of collaborating with genealogical organizations and ensure that the data are not locked behind proprietary firewalls.

## Footnotes

1 In addition to the United States, during this period we also worked directly or indirectly with genealogical organizations in Britain, Canada, Denmark, Iceland, Ireland, Norway, and Sweden. In most of those countries, we had academic partners who negotiated the terms of collaboration with each genealogical organization (Roberts et al., 2003; Ruggles et al., 2011). The first digital transcription of a complete individual-level historical census began in 1968, when Jan Oldervoll launched a project to transcribe the entire 1801 census of Norway (Thorvaldsen & Solli, 2012).

2 A secondary reason to obtain digital images was to explore the potential for machine-assisted transcription through automated handwriting recognition. After exploring the state of technology with investigators working on handwriting recognition, we decided that it was not yet a cost-effective option.
